# Novel chlorinated and nitrogenated azaphilones with cytotoxic activities from the marine algal-derived fungus *Chaetomium globosum* 2020HZ23

**DOI:** 10.3389/fmicb.2023.1252563

**Published:** 2023-08-21

**Authors:** Zhong-Jie Gao, Lu-Lu Cao, Hai-Ping Ren, Hua Yu, Yan Wang

**Affiliations:** Qingdao Hiser Hospital Affiliated of Qingdao University (Qingdao Traditional Chinese Medicine Hospital), Qingdao, China

**Keywords:** azaphilones, secondary metabolites, marine fungus, *Chaetomium globosum*, cytotoxic activity

## Abstract

Two novel chlorinated and nitrogenated azaphilones, namely *N*-butyl-2-aza-2-deoxychaetoviridin A (**1**) and *N*-hexyl-2-aza-2-deoxychaetoviridin A (**2**), along with a previously identified analogue, chaetoviridin A (**3**), were successfully obtained from *Chaetomium globosum* 2020HZ23, a marine algal-sourced endophytic fungus. The planar structures as well as the absolute configurations of these new metabolites were determined utilizing a synergistic approach that involved both spectroscopic techniques (1D/2D NMR and HRESIMS) and Density Functional Theory (DFT) calculations. Each compound was subject to *in vitro* cytotoxicity evaluation toward the A549 cancer cell line. Both compounds **1** and **2** demonstrated significant cytotoxicity, as evidenced by their respective IC_50_ values of 13.6 and 17.5 μM. Furthermore, **1** and **2** demonstrated potent cell migration inhibition, which elevated with increasing dose concentration. In contrast, compound **3** exhibited less cytotoxic activity relative to **1** and **2**, suggesting that the cytotoxic potency escalates with N-substitution at the C-2 position and the introduction of a side chain. This finding could offer implications for future studies aimed at designing and refining lead compounds within this class.

## 1. Introduction

Azaphilones, predominantly originating from fungi, especially the Ascomycetes phylum, are an assembly of naturally occurring fungal polyketide metabolites (Zeng et al., 2023). With a highly oxygenated and bicyclic core structure, they are accented with various functional groups. Their core structure includes two features, a pyranoquinone bicyclic component known as isochrome and a quaternary chiral center of *R* or *S* stereochemistry (Gao et al., 2013; Wang et al., 2018; Chen et al., 2020; Wang et al., 2020). Exhibiting considerable structural diversity, azaphilones are characterized by modifications made to their core structures and variations in side-chain substitutions (Chen et al., 2016). While some azaphilones possess elaborate side chains that diversify the core skeleton, others introduce modified nuclei *via* alterations in the chromane-quinone methide structure (Li et al., 2014). The structural versatility inherent to azaphilones equips them with a wide array of biological functionalities, encompassing antimicrobial, antifungal, cytotoxic, and anti-inflammatory activities among others. This wide-ranging biological activity profile accentuates their potential applicability within the realms of antiviral and anticancer therapeutics (Pavesi et al., 2021).

As belonging to the Chaetomiaceae family, the genus *Chaetomium* with over 400 species has emerged as an important reservoir for novel bioactive metabolites. Long-term chemistry studies on *Chaetomium* species have shed light on the extensive structural diversity and remarkable bioactivity potential of specialized metabolites. To date, this genus has reported to produce over 500 unique natural compounds, inclusive of azaphilones, cytochalasans, pyrones, alkaloids, diketopiperazines, anthraquinones, polyketides, and steroids (Rao et al., 2023). For example, a bioassay-guided isolation of the endophytic *C. globosum* yielded twelve specialized metabolites, including six azaphilones (Qi et al., 2020). Chaetomugilins D and J, azaphilone derivatives isolated from the same species, displayed suppression of lettuce seed germination and inhibited root and shoot growth, hinting at their herbicidal capabilities (Piyasena et al., 2015). Nitrogenous azaphilones, sourced from indoor air-derived fungus *C. globosum* DAOM 240359, displayed antibacterial properties against *Pseudomonas putida* and *Bacillus subtilis* (McMullin et al., 2013). Chaetomugilides A − C, along with three known compounds isolated from *C. globosum* TY1, demonstrated cytotoxic behavior against the HepG2 cancer cell line (Li et al., 2013). Two previously unknown azaphilone alkaloid dimers, chaetofusins A and B, were isolated from the endophytic fungus *C. fusiforme* obtained from liverwort (Peng et al., 2012).

**Figure 1:**
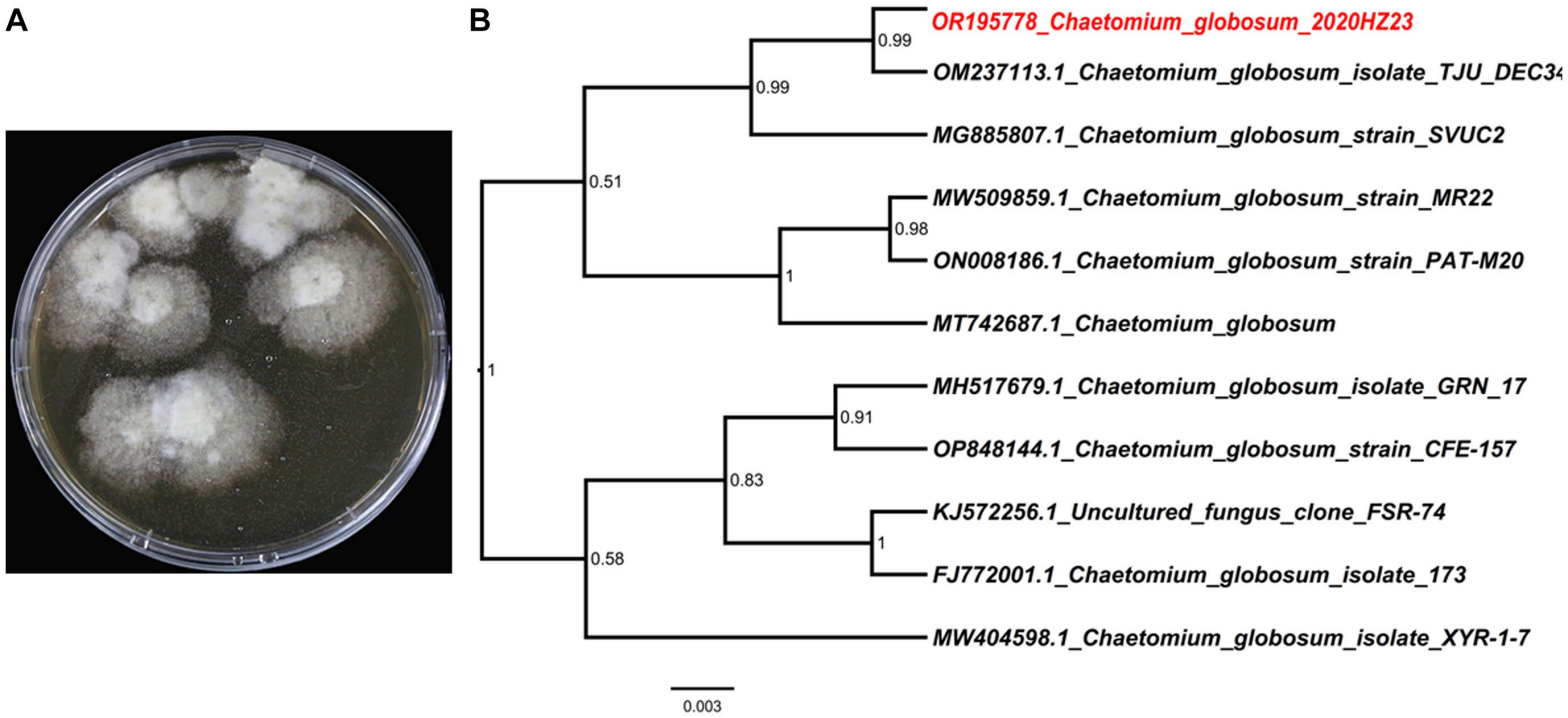
Morphology of *Chaetomium globosum* 2020HZ23 on PDA medium **(A)** and phylogenomic tree of *C. globosum* 2020HZ23 **(B)**.

**Figure 2:**
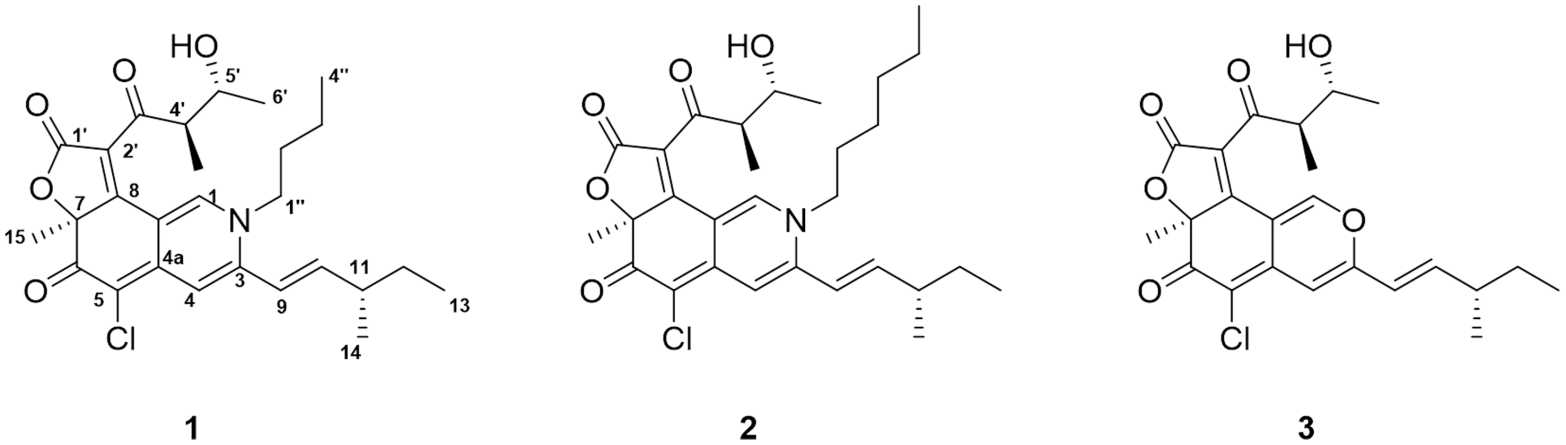
Structures of compounds **1–3.**

Among the reported azaphilones, chaetoviridins stand out as a distinct subclass, synthesized by the *Chaetomium* genus of fungi (Yang et al., 2021). In 1990, Takahashi et al. (1990) firsly elucidated the structure of chaetoviridin A featuring the (4’*S*, 5’*R*) syn aldol side chain, and then subsequently facilitated the structural assignment of other epimers. Makrerougras et al. (2017), however, subsequently modified the stereochemistry at the C-4′ and C-5′ positions of chaetoviridin A to (4’*R*, 5’*R*), achieved *via* the complete synthesis of (4’*R*, 5’*R*)-chaetoviridin A and its associated epimers. As a part of an ongoing pursuit for bioactive natural compounds from marine-derived fungi, *Chaetomium globosum* 2020HZ23 (Figure 1) was unearthed as an endophyte of the marine brown algae, *Sargassum thunbergii*. Utilizing a combination of spectroscopic methods as well as DFT calculation, the characterization of three distinct secondary metabolites was carried out. As a result, two new azaphilones, namely *N*-butyl-2-aza-2-deoxychaetoviridin A (**1**) and *N*-hexyl-2-aza-2-deoxychaetoviridin A (**2**), alongside the chaetoviridin A (**3**) with a biogenetic relation, were characterized (Figure 2). The new compounds were then subjected to cytotoxic activity testing toward the A549 cancer cell line to evaluate their potential use as anticancer agents. The results indicated that the new azaphilones **1** and **2** demonstrated significant cytotoxicity and inhibited cell migration. Herein we report the isolation, structural elucidation, and cytotoxic assessment of the newly-discovered azaphilones.

## 2. Materials and methods

### 2.1. General experimental procedures

For open column chromatography applications, silica gel of mesh sizes 100–200 and 200–300 (Qingdao Marine Chemical Inc., Qingdao, China), Lobar LiChroprep RP-18 (40–60 μm, Merck, Darmstadt, Germany), and Sephadex LH-20 (Merck) were the materials of choice. High-Resolution Electrospray Ionization Mass Spectrometry (HRESIMS) experiments, conducted in positive ion mode, utilized a Waters Xevo G2-XS QTof mass spectrometer (Waters, Milford, MA, United States). Nuclear Magnetic Resonance (NMR) spectroscopic data were collected with a Bruker Avance 600 MHz spectrometer, employing tetramethylsilane (TMS) as an internal standard for calibration. Optical rotations were ascertained utilizing an MCP 500 polarimeter instrument manufactured by Anton Paar. Ultraviolet (UV) spectroscopic analyses were carried out on a Shimadzu UV-1800 spectrometer (Shimadzu Co., Ltd., Kyoto, Japan).

### 2.2. Fungal source

*C. globosum* 2020HZ23, the producing fungal strain, was originally separated from the inner tissues of the marine brown algae *Sargassum thunbergii*, harvested in September 2020 from Qingdao, China. Morphological attributes along with sequencing of the Internal Transcribed Spacer (ITS) region (GenBank accession no. OR195778) (Figure 1) facilitated the precise identification of this strain as *C. globosum* 2020HZ23. To clearly indicate the evolutionary position of this fungal strain *C. globosum* 2020HZ23, a phylogenetic analysis based on its ITS sequence as well as those from other *Chaetomium* species, was performed. As shown in Figure 1, the fungus *C. globosum* 2020HZ23 was located at the head position of the entire phylogenomic tree with a high confidence of 99%. This fungus is currently deposited at the Qingdao Hiser Hospital Affiliated of Qingdao University.

### 2.3. Process of fermentation, extraction, and isolation

The fungal strain underwent fermentation in a static state on a solid rice medium. Each 1 L Erlenmeyer flask contained a concoction of 0.1 g sodium glutamate, 0.1 g corn flour, 0.3 g peptone, 1 g mannitol, 1 g maltose, 2 g D-glucose, 70 g rice, and 100 mL seawater from Qingdao Beach. The pH value was regulated to 6.5 prior to the fermentation process, which lasted 25 days at ambient temperature. Post fermentation, methanol extraction of the liquid was executed, followed by a triple filtration with Whatman filter paper. The methanolic extract was then concentrated under reduced pressure and partitioned between water and ethyl acetate. Further vacuum concentration of the ethyl acetate fraction yielded a 150 g extract. The extract was subjected to silica gel column chromatography with a gradient of petroleum ether and ethyl acetate to yield five fractions (Frations 1–5), which were consolidated based on thin-layer chromatography (TLC) analyses. Fraction 1 was further purified with Sephadex LH-20, producing compound **2** (2.3 mg) and compound **1** (1.8 mg). Fraction 2 was processed through Sephadex LH-20 and reversed-phase HPLC (20–50% MeCN/H_2_O, with the timespan of 10.0 min and the flow rate of 10 mL/min), yielding compound **3** (2.0 mg, *t*_R_ = 6.413 min).

Compound **1**: A red amorphous powder; [α]^25^_D_ + 1,270 (*c* 0.005, MeOH); UV (MeOH) *λ*_max_ (log *ε*): 225 (4.15), 295 (4.12) nm; ECD (*c* 1.0 mg/mL, MeOH) *λ*_max_ (∆*ε*): 230 (−22.6), 310 (+28.2), 380 (−22.1); ^1^H and ^13^C NMR data, shown in Table 1; HRESIMS *m/z* 510.2022 [M + Na]^+^ (calcd for C_27_H_34_ClNO_5_Na, 510.2022).

**Table 1:** NMR Data for Compounds 1 and 2 in Chloroform-*d.*

No	**1**		No	**2**	
	*δ_C_*, type	*δ_H_* (*J* in Hz)		*δ_C_*, type	*δ_H_* (*J* in Hz)
1	141.5, CH	8.72, s	1	141.6, CH	8.72 (s, 1H)
3	147.2, C	–	3	147.3, C	–
4	111.2, CH	6.88, s	4	111.3, CH	6.90 (s, 1H)
4a	144.9, C	–	4a	145.0, C	–
5	99.9, C	–	5	99.9, C	–
6	181.5, C	–	6	181.1, C	–
7	88.9, C	–	7	88.8, C	–
8	168.8, C	–	8	168.8, C	–
8a	111.4, C	–	8a	111.5, C	–
9	119.1, CH	6.22, d (15.4)	9	119.1, CH	6.24, d (15.4)
10	149.3, CH	6.42, dd (15.4,7.9)	10	149.4, CH	6.43, dd (15.4, 7.9)
11	39.4, CH	2.32, m	11	39.4, CH	2.32, m
12	29.1, CH_2_	1.48, m	12	29.1, CH_2_	1.49, m
13	11.8, CH_3_	0.94, t (7.4)	13	11.8, CH_3_	0.95, t (7.4)
14	19.4, CH_3_	1.12, d (6.7)	14	19.4, CH_3_	1.13, d (6.7)
15	27.4, CH_3_	1.71, s	15	27.4, CH_3_	1.70, s
1’	168.8, C	–	1’	168.6, C	–
2’	123.0, C	–	2’	123.1, C	–
3’	201.8, C	–	3’	201.8, C	–
4’	50.8, CH	3.70, qd (6.8, 6.6)	4’	50.8, CH	3.71, qd (6.8, 6.6)
5’	70.8, CH	3.86, qd (6.5, 6.6)	5’	70.8, CH	3.87, qd (6.5, 6.6)
6’	21.3, CH_3_	1.14, d (6.5)	6’	21.3, CH_3_	1.14, d (6.5)
7’	13.7, CH_3_	1.18, d (6.8)	7’	13.6, CH_3_	1.18, d (6.8)
1”	54.4, CH_2_	3.93, t (7.5)	1”	54.8, CH_2_	3.95, t (7.5)
2”	32.1, CH_2_	1.80, m	2”	30.1, CH_2_	1.82, m
3”	19.6, CH_2_	1.44, m	3”	26.0, CH_2_	1.41, m
4”	13.6, CH_3_	1.02, t (7.4)	4”	31.2, CH_2_	1.35, m
			5”	22.4, CH_2_	1.35, m
			6”	13.9, CH_3_	0.92, t (7.4)

Compound **2**: A red amorphous powder; [α]^25^_D_ + 1,360 (*c* 0.005, MeOH); UV (MeOH) *λ*_max_ (log *ε*): 220 (4.24), 295 (4.18) nm; ECD (*c* 1.0 mg/mL, MeOH) *λ*_max_ (∆*ε*): 230 (−30.2), 310 (+25.2), 375 (−24.2); ^1^H and ^13^C NMR data, shown in Table 1; HRESIMS *m/z* 538.2333 [M + Na]^+^ (calcd for C_29_H_38_ClNO_5_Na, 538.2336).

### 2.4. Computational details

Candidate conformers were generated utilizing the Conformer Rotamer Ensemble Sampling Tool (CREST) (Grimme, 2019; Pracht et al., 2020) and subject to Density Functional Theory (DFT) calculations *via* the Gaussian 16 program (Frisch et al., 2016). Conformers that fell within a 10 kcal/mol energy window were optimized at the B3LYP/6-31G(d) level of theory, implementing Grimme’s D3 dispersion correction. All optimized conformations underwent frequency analysis at the identical theoretical level to ascertain their local minima status on the potential energy surface. The energy values of all optimized conformations were then determined using the M062X/6–311 + G(2d,p) level with D3 dispersion correction. By combining the “Thermal correction to Gibbs Free Energy” from frequency analysis with electronic energies from M062X/6–311 + G(2d,p), Gibbs free energies were calculated for each conformer. Utilizing the Boltzmann distribution law, equilibrium populations at room temperature (298.15 K) were determined. Conformers with population values above 2% underwent additional computations. Electronic Circular Dichroism (ECD) Time-dependent Density Functional Theory (TDDFT) calculations were executed at the CAM-B3LYP/6-311G(d) level of theory, in methanol (MeOH) and with the application of the IEFPCM solvent model. 36 excited states were computed for each conformer (Pescitelli and Bruhn, 2016). The resultant ECD curves were developed using the Multiwfn 3.6 software (Lu and Chen, 2012).

### 2.5. Cell lines and reagents

A549 cells were purchased from National Collection of Authenticated Cell Cultures (China) and incubated with RPMI 1640 (Gibco, Beijing, China) with 10% fetal bovine serum (FBS; Gibco). Cells were grown at 37°C in a 5% CO_2_ humidified atmosphere.

### 2.6. Cell viability assays

Cell viability was assessed using the Cell Counting Kit-8 (CCK-8, MCE, United States) according to the guidance of the manufacturer. Cells were seeded into 96-well plates and collected after treatment for 48 h. 10 μL of CCK-8 solution was added into the cultures at 37°C for 1 h. The absorbance at 450 nm was measured with a microplate reader (Spark multimode microplate reader, Tecan, Austria).

### 2.7. Wound healing assay

A549 cells were seeded in six-well plates until cell confluence reached approximately 100%. The wounds were scratched with 10 μL pipette tips, and cells were washed with PBS. The cells were cultured with 1% FBS medium. The scratch recovery was observed at 0 and 48 h, and the healing rates were estimated with ImageJ software.

## 3. Results and discussion

### 3.1. Structural elucidation

**Figure 3:**
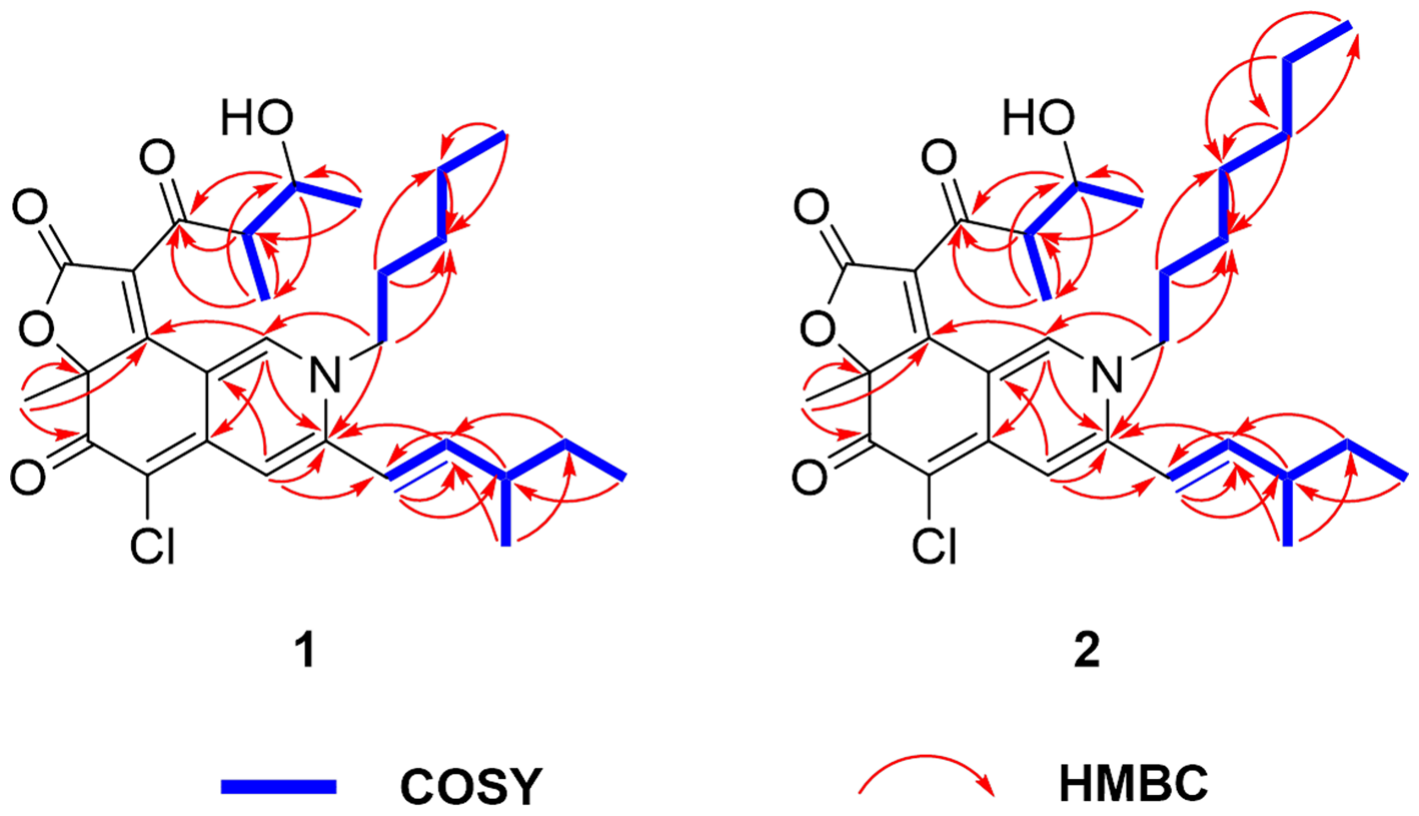
Selected COSY and HMBC correlations of compounds **1** and **2.**

**Figure 4:**
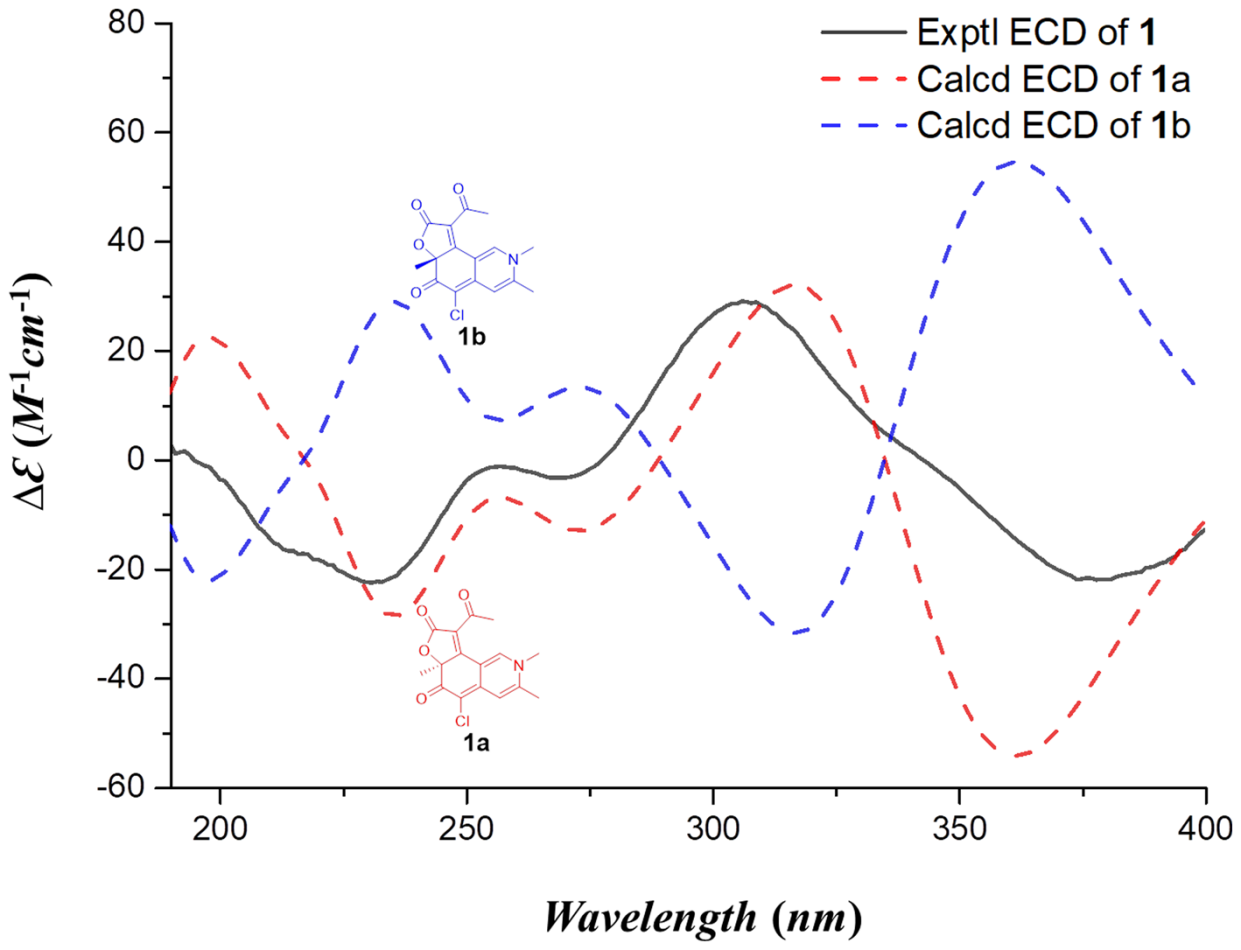
Experimental and calculated ECD spectra of compound **1.**

**Figure 5:**
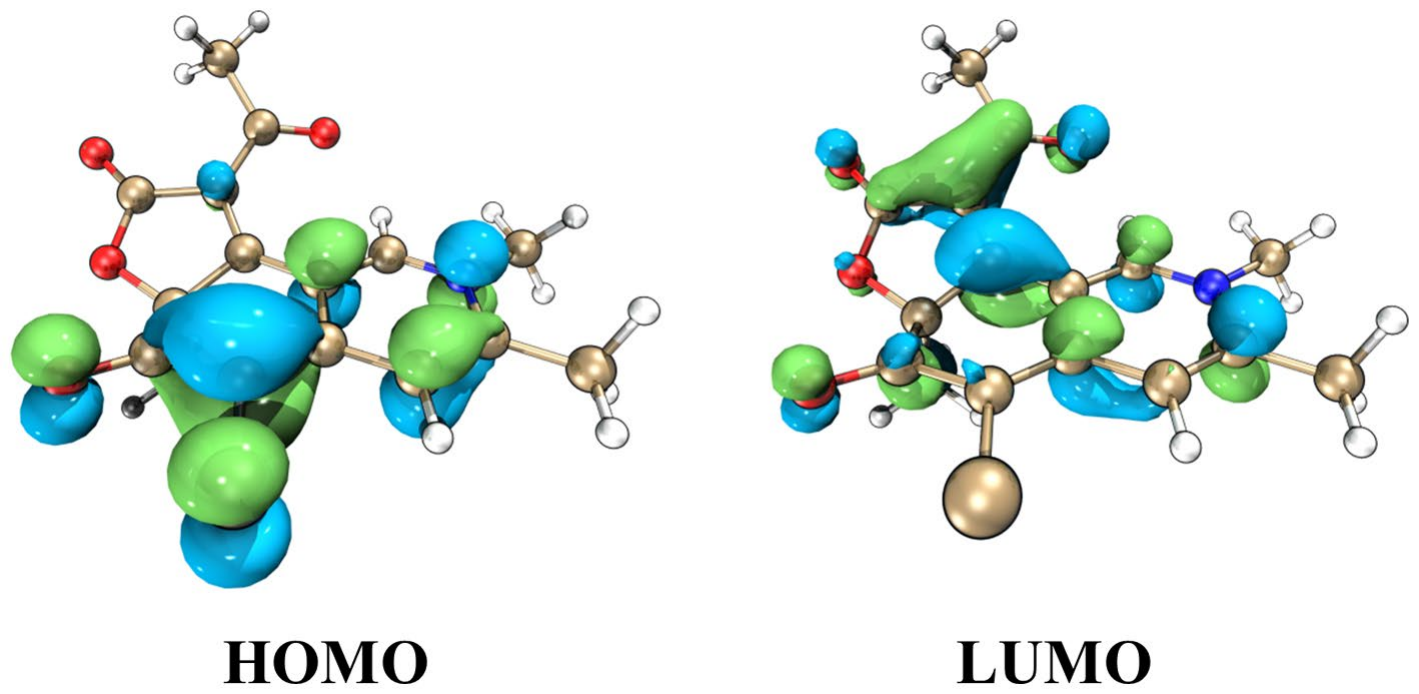
Key molecular orbitals (MOs) involved in the important transitions of **1** and **2** regarding ECD spectra of conformer **1a** in the gas phase at the B3LYP/6-31G(d) level.

The molecular formula of Compound **1**, a dark red solid, is confirmed as C_27_H_34_ClNO_5_ through High-Resolution Electrospray Ionization Mass Spectrometry (HRESIMS), suggesting 11 degrees of unsaturation. The chlorine atom was authenticated through the isotopic peak observation of [M + H]^+^: [M + H + 2]^+^ at a 3:1 ratio. ^13^C, DEPT, and HSQC spectra revealed one disubstituted double bond (*δ*_C_ 119.1 and 149.3), two trisubstituted double bonds (*δ*_C_ 147.2 and 111.2; *δ*_C_ 141.5 and 111.4), two tetrasubstituted double bonds (*δ*_C_ 144.9 and 99.9; *δ*_C_ 123.0 and 168.8), one ester carbonyl carbon (*δ*_C_ 168.8), and two keto carbons (*δ*_C_ 181.5 and 201.8) (Table 1). Structural similarities between **1** and the co-isolated chaetoviridin A (**3**) were noted from the spectral data, with both compounds possessing a tricyclic core with two side chains, an aldol group and a methyl-branched pentenyl. The most noticeable differences were observed in the chemical shifts of C-1 (from *δ*_C_ 151.6 in **3** to *δ*_C_ 141.5 in **1**) and C-3 (from *δ*_C_ 157.0 in **3** to *δ*_C_ 147.2 in **1**). Moreover, four extra resonances in compound **1**, indicative of a butyl unit, were observed. Given the chemical shifts for C-1 and C-3 and the overall molecular weight, a nitrogen atom was postulated at the 2 position, bearing a butyl group. This inference was supported by COSY correlations of H_2_-1”/H_2_-2”/H_2_-3”/H_3_-4″ and HMBC correlations of H_2_-1″ with C-1 and C-3 (Figure 3). The *E* configuration of the C-9-C-10 double bond was determined *via* ^3^*J*_H9-H10_ (15.4) (Table 1). The absolute configurations at C-4′, C-5′, and C-11 were established as 4’*R*, 5’*R*, 11*S*, based on NMR data comparison with the co-isolated **3**, as well as the four previously synthesized chaetoviridin A epimers (Makrerougras et al., 2017), considering the same biosynthetic pathway. To determine the stereochemistry of C-7, we conducted ECD calculations on the simplified structures of (7*S*)-**1** (**1a**) and (7*R*)-**1** (**1b**), which resulted in the assignment of the C-7 position as *S* (Figure 4). Additionally, the negative CE at approximately 380 nm was attributed to the electron transition from MO83 (HOMO) to MO84 (LUMO) (Figure 5), in alignment with the ECD spectrum of chaetoviridin A reported by Steyn and Vleggaar (1976), as well as the nitrogenated azaphilones reported by Wang et al. (2020). Consequently, we identified the compound **1** as *N*-butyl-2-aza-2-deoxychaetoviridin A.

Compound **2**, procured as a dark red solid, was assigned a molecular formula of C_29_H_38_ClNO_5_ through HRESIMS analysis. The chlorine atom was also authenticated through the isotopic peak observation of [M + H]^+^:[M + H + 2]^+^ at a 3:1 ratio. Additionally, ^13^C, DEPT, and HSQC spectra were utilized, which revealed the presence of one disubstituted double bond (*δ*_C_ 119.1 and 149.4), two trisubstituted double bonds (*δ*_C_ 147.3 and 111.3; *δ*_C_ 141.6 and 111.5), two tetrasubstituted double bonds (*δ*_C_ 145.0 and 99.9; *δ*_C_ 123.1 and 168.6), one ester carbonyl carbon (*δ*_C_ 168.6), and two keto carbons (*δ*_C_ 181.1 and 201.8) (Table 1). Structural similarities between **2** and the co-isolated **3** were observed in the spectral data. Both compounds possess a tricyclic core with two side chains, an aldol group, and a methyl-branched pentenyl. Upon comparing the NMR data of **2** to that of **1**, it was noted that they share the same stereogenic centers, while differences manifest in the side chain attached to N-2. The presence of two additional carbon resonances compared to **1**, along with COSY correlations of H_2_-1”/H_2_-2’/H_2_-3”/H_2_-4”/H_2_-5”/H_3_-6″ and HMBC correlations of H_2_-1”/C-1 and H_2_-1”/C-3, confirmed the attachment of a hexyl group to N-2 (Figure 3). Consequently, following further 2D NMR analysis, the structure of **2** was ascertained to be *N*-hexyl-2-aza-2-deoxychaetoviridin A.

Compound **3** was ascertained as chaetoviridin A through the comparison of NMR data with those documented in existing literature (Park et al., 2005).

### 3.2. Cytotoxic activity

**Figure 6:**
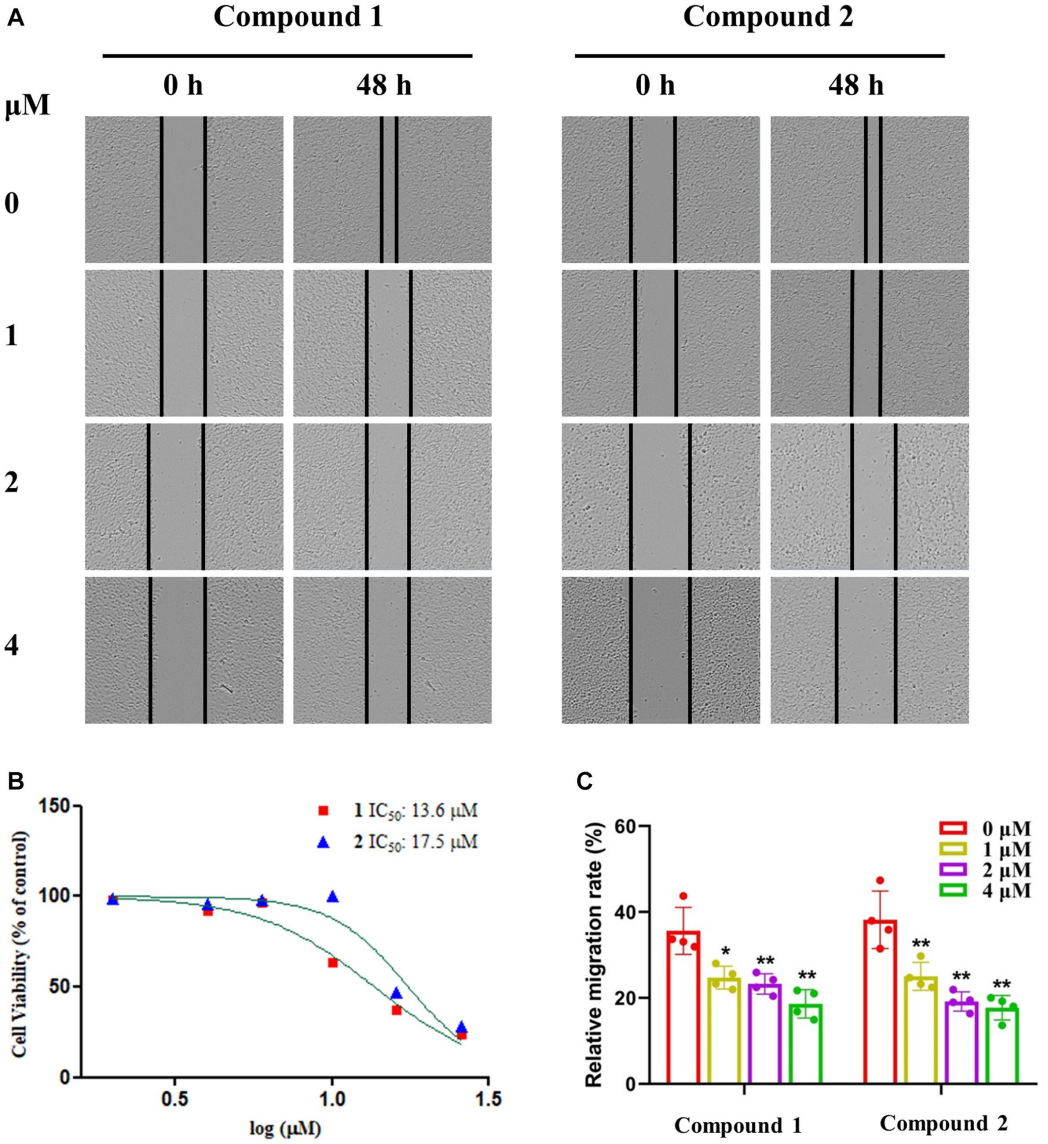
Compounds **1** and **2** exhibited inhibitory effects on both proliferation and migration in the A549 cell line. **(A,C)** The wound healing assay, used to assess cell migration, was executed on the A549 cell line following treatment with compounds **1** and **2** at the specified concentrations for 48 h. Statistical significance was indicated by ^*^ *p <* 0.05; ^**^p < 0.01. **(B)** A549 cell line was treated with defined concentrations of compounds **1** and **2** for specific time durations. The viability of cells was quantified using a CCK8 assay.

We utilized a CCK8 assay to examine the impact of compounds **1**–**3** on the viability of the A549 cancer cell line. Both compounds **1** and **2** demonstrated dose-dependent cytotoxicity, with IC_50_ values of 13.6 and 17.5 μM (Figure 6B), respectively, while compound **3** showed a low degree of cytotoxicity (IC_50_ > 50 μM, data were not shown). The cytotoxic results suggested that the cytotoxic potency escalates with N-substitution at the C-2 position and the introduction of a side chain. A wound-healing assay was conducted to assess the impact of compounds **1** and **2** on the migration and invasion capabilities of A549 cells. Figures 6A,C illustrate that A549 cells in the control group were able to migrate across the complete wound area within a 48-h period. However, cell migration was significantly curtailed in a dose-dependent manner when treated with specific concentrations (1, 2 and 4 μM) of compounds **1** and **2**.

## 4. Conclusion

Marine-derived fungal secondary metabolites are garnering increased attention owing to their distinctive structural properties and potent pharmacological possibilities. Within this field of study, the current investigation has yielded two novel nitrogenated azaphilones, *N*-butyl-2-aza-2-deoxychaetoviridin A (**1**) and *N*-hexyl-2-aza-2-deoxychaetoviridin A (**2**), along with the previously identified azaphilone chaetoviridin A (**3**). These compounds were derived from the solid culture of the marine fungus *Chaetomium globosum* 2020HZ23. By employing a combination of spectroscopic techniques and DFT calculations, the absolute configurations of compounds **1** and **2** were determined. Additionally, the isolated compounds underwent cytotoxicity evaluations, uncovering their cytotoxic effects on the A549 cell line. Compound **1** displayed an IC_50_ value of 13.6 μM, whereas compound **2** exhibited an IC_50_ value of 17.5 μM. Furthermore, both compounds demonstrated a dose-dependent inhibition of cell migration. In contrast, compound **3** presented lower cytotoxic activity compared to compounds **1** and **2**, indicating that cytotoxicity intensifies with the incorporation of *N*-substitution at the 2 position and the addition of a side chain. This observation could prove instrumental for future research focused on the design and optimization of lead compounds within this category.

## Data availability statement

The original contributions presented in the study are included in the article/Supplementary material, further inquiries can be directed to the corresponding authors.

## Author contributions

Z-JG and L-LC: experiment implementation and writing—original draft preparation. Z-JG, L-LC, and H-PR: data analysis. HY and YW: writing—review and editing. All authors contributed to the article and approved the submitted version.

## Conflict of interest

The authors declare that the research was conducted in the absence of any commercial or financial relationships that could be construed as a potential conflict of interest.

## Publisher’s note

All claims expressed in this article are solely those of the authors and do not necessarily represent those of their affiliated organizations, or those of the publisher, the editors and the reviewers. Any product that may be evaluated in this article, or claim that may be made by its manufacturer, is not guaranteed or endorsed by the publisher.
