# Supplementary material for: Novel chlorinated and nitrogenated azaphilones with cytotoxic activities from the marine algal-derived fungus Chaetomium globosum 2020HZ23
Source: Front Microbiol. 2023 Aug 21;14:1252563. doi: 10.3389/fmicb.2023.1252563 (PMC10475718; doi:10.3389/fmicb.2023.1252563)
Supplement: Supplementary file 1 [file Data_Sheet_1.docx]

Supplementary Material

Novel chlorinated and nitrogenated azaphilones with cytotoxic activities from the marine algal-derived fungus *Chaetomium* *globosum* 2020HZ23

Zhong-Jie Gao^1,#^, Lu-Lu Cao^1,#^, Hai-Ping Ren^1^, Hua Yu^1,^*, Yan Wang^1,^*

^1^Qingdao Hiser Hospital Affiliated of Qingdao University (Qingdao Traditional Chinese Medicine Hospital), Qingdao 266033, China

Table of Content

[Figure S1. CNMR spectrum of 1 2](#_Toc138952561)

[Figure S2. HNMR spectrum of 1 2](#_Toc138952562)

[Figure S3. DEPT spectrum of 1 3](#_Toc138952563)

[Figure S4. HSQC spectrum of 1 3](#_Toc138952564)

[Figure S5. HMBC spectrum of 1 4](#_Toc138952565)

[Figure S6. COSY spectrum of 1 4](#_Toc138952566)

[Figure S7. MS spectrum of 1 5](#_Toc138952567)

[Figure S8. CNMR spectrum of 2 5](#_Toc138952568)

[Figure S9. HNMR spectrum of 2 6](#_Toc138952569)

[Figure S10. DEPT spectrum of 2 6](#_Toc138952570)

[Figure S11. HSQC spectrum of 2 7](#_Toc138952571)

[Figure S12. HMBC spectrum of 2 7](#_Toc138952572)

[Figure S13. COSY spectrum of 2 8](#_Toc138952573)

[Figure S14. MS spectrum of 2 8](#_Toc138952574)

[Table S1. Conformational analysis of the B3LYP/6-31G(d) optimized conformers of 1a in the gas phase (T=298.15 K) 8](#_Toc138952575)

[Table S2. Key transitions, oscillator strengths, and rotatory strengths in the ECD spectrum of conformer cpd-1a-1 at the Cam-B3LYP/6-311G(d) level of theory in MeOH with IEFPCM solvent model. 9](#_Toc138952576)

1. CNMR spectrum of **1**

1. HNMR spectrum of **1**

1. DEPT spectrum of **1**

1. HSQC spectrum of **1**

1. HMBC spectrum of **1**

1. COSY spectrum of **1**


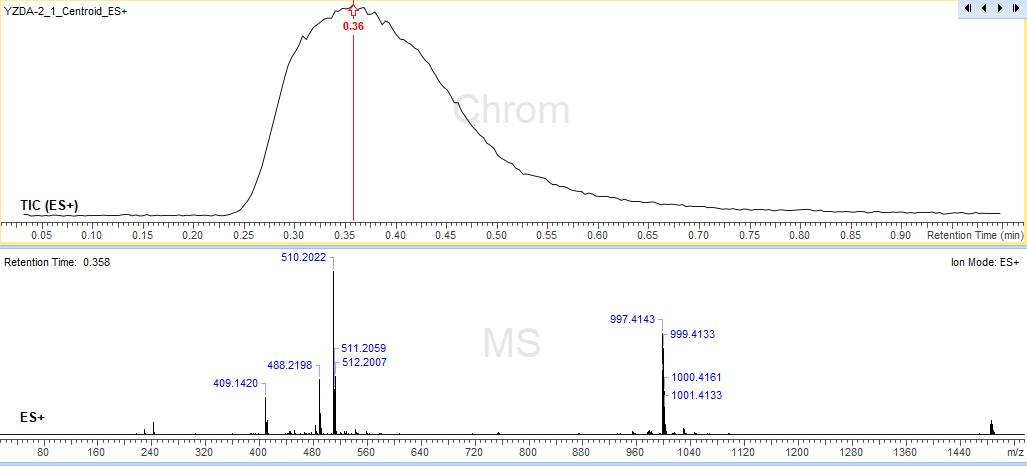


1. MS spectrum of **1**

1. CNMR spectrum of **2**

1. HNMR spectrum of **2**

1. DEPT spectrum of **2**

1. HSQC spectrum of **2**

1. HMBC spectrum of **2**

1. COSY spectrum of **2**


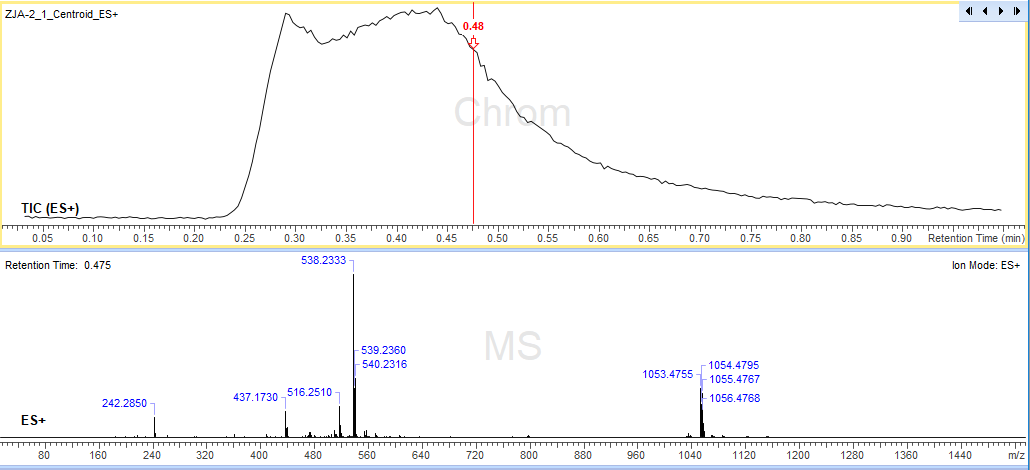


1. MS spectrum of **2**
2. Conformational analysis of the B3LYP/6-31G(d) optimized conformers of **1a** in the gas phase (T=298.15 K)

| **Conformer** | **E*^a^* (Hartree)** | **C*^b^* (Hartree)** | **G*^c^* (kcal/mol)** | **ΔG*^d^* (kcal/mol)** | **Population*^e^*** |
| --- | --- | --- | --- | --- | --- |
| **1a-1** | -1433.887125 | 0.220407 | -899625.865683 | 0.0 | 100.00% |

*^a^*Electronic energy obtained at M062X/6-311+G(2d,p) level of theory; *^b^*Thermal correction to Gibbs free energy obtained at B3LYP/6-31G(d) level of theory; *^c^*Gibbs free energy (E + C); *^d^*The relative Gibbs free energy; *^e^*The Boltzmann distribution of each conformer.

Table @@@. Atomic coordinates (Å) of **1a-1** obtained at the B3LYP/6-31G(d) level of theory in the gas phase.

| C | -0.519075 | 2.309841 | -0.091814 | O | -1.394630 | -3.124398 | -0.194143 |
| --- | --- | --- | --- | --- | --- | --- | --- |
| C | -1.324335 | 1.224770 | 0.669133 | C | -3.483487 | -2.636705 | -1.231018 |
| C | -0.939364 | -0.179021 | 0.289002 | C | 4.686376 | -1.266288 | -0.131734 |
| C | 0.479437 | -0.449524 | 0.220461 | C | 2.811689 | -3.341137 | 0.446767 |
| C | 1.364362 | 0.676540 | -0.077760 | H | 0.352991 | -2.546748 | 0.546736 |
| C | 0.874873 | 1.953137 | -0.277018 | H | 3.464262 | 1.119705 | -0.416288 |
| C | 1.008799 | -1.706640 | 0.364411 | H | -0.058995 | 1.381278 | 2.445778 |
| N | 2.339880 | -1.967749 | 0.259173 | H | -1.456342 | 2.486558 | 2.408639 |
| C | 3.227653 | -0.932217 | -0.024082 | H | -1.701473 | 0.751962 | 2.750429 |
| C | 2.756193 | 0.332808 | -0.192998 | H | -3.412100 | -3.683188 | -1.532115 |
| O | -2.720072 | 1.326121 | 0.400134 | H | -4.374824 | -2.467390 | -0.619604 |
| C | -3.212444 | 0.102281 | 0.033391 | H | -3.599533 | -1.991747 | -2.108766 |
| C | -2.079011 | -0.865942 | -0.020692 | H | 4.883095 | -1.973370 | -0.946454 |
| Cl | 1.935309 | 3.247367 | -0.805925 | H | 5.256198 | -0.357114 | -0.330483 |
| O | -1.030344 | 3.382606 | -0.359556 | H | 5.071902 | -1.714226 | 0.791661 |
| C | -1.114007 | 1.474889 | 2.176788 | H | 3.358416 | -3.685857 | -0.434826 |
| O | -4.385380 | -0.072709 | -0.194009 | H | 3.462666 | -3.415536 | 1.323027 |
| C | -2.242311 | -2.270536 | -0.453562 | H | 1.945635 | -3.985824 | 0.593696 |

1. Key transitions, oscillator strengths, and rotatory strengths in the ECD spectrum of conformer cpd-**1a-1** at the Cam-B3LYP/6-311G(d) level of theory in MeOH with IEFPCM solvent model.

| ***Num^a^*** | ***Transition^b^*** | ***CI-coeff^b^*** | ***ΔE (eV)^d^*** | ***λ (nm)^e^*** | ***f^f^*** | ***R_vel_^g^*** | ***R_len_^h^*** |
| --- | --- | --- | --- | --- | --- | --- | --- |
| 1 | 83->84 | 0.6952 | 2.7642 | 448.54 | 0.0918 | 77.9926 | 77.3407 |
| 2 | 80->84 | 0.38393 | 3.8807 | 319.49 | 0.0188 | 50.5704 | 52.7894 |
|  | 80->86 | -0.22817 |  |  |  |  |  |
|  | 81->84 | 0.41851 |  |  |  |  |  |
| 3 | 80->84 | -0.24164 | 3.9290 | 315.56 | 0.3603 | -296.8566 | -303.213 |
|  | 83->85 | 0.60012 |  |  |  |  |  |
| 4 | 82->84 | 0.48892 | 4.1502 | 298.75 | 0.1267 | 193.681 | 196.6902 |
|  | 82->85 | -0.29633 |  |  |  |  |  |
|  | 83->85 | 0.27826 |  |  |  |  |  |
| 5 | 80->84 | -0.33766 | 4.7438 | 261.36 | 0.4545 | 22.6711 | 24.2032 |
|  | 81->84 | 0.40409 |  |  |  |  |  |
|  | 82->84 | 0.31274 |  |  |  |  |  |
| 6 | 83->86 | 0.52727 | 4.9907 | 248.43 | 0.0280 | 18.4898 | 19.6687 |
|  | 83->87 | -0.32126 |  |  |  |  |  |
| 7 | 79->84 | 0.53508 | 5.0399 | 246.01 | 0.0289 | -45.0962 | -45.2714 |
| 8 | 79->84 | -0.26627 | 5.2583 | 235.79 | 0.1654 | -12.9615 | -12.8638 |
|  | 81->84 | -0.31245 |  |  |  |  |  |
|  | 82->85 | 0.38975 |  |  |  |  |  |
| 9 | 81->85 | 0.37333 | 5.6072 | 221.12 | 0.0159 | -4.6021 | -7.0422 |
|  | 83->86 | 0.2527 |  |  |  |  |  |
|  | 83->88 | -0.29452 |  |  |  |  |  |
| 10 | 81->85 | 0.32386 | 5.6488 | 219.49 | 0.0390 | -6.8358 | -7.0681 |
|  | 83->88 | 0.45876 |  |  |  |  |  |
|  | 83->90 | 0.24961 |  |  |  |  |  |
| 11 | 81->86 | -0.25009 | 5.8067 | 213.52 | 0.0322 | 53.6313 | 55.0536 |
|  | 83->87 | 0.46193 |  |  |  |  |  |
| 12 | 77->84 | 0.27021 | 5.9476 | 208.46 | 0.0332 | -74.3153 | -77.0297 |
|  | 80->86 | 0.33668 |  |  |  |  |  |
|  | 83->87 | 0.2823 |  |  |  |  |  |
| 13 | 78->84 | 0.59341 | 6.0464 | 205.06 | 0.0486 | 29.2607 | 29.5512 |
| 14 | 75->84 | -0.25521 | 6.0775 | 204.00 | 0.0218 | -36.2693 | -37.1833 |
|  | 77->84 | 0.48255 |  |  |  |  |  |
| 15 | 75->84 | 0.25289 | 6.3927 | 193.95 | 0.0517 | -16.1629 | -15.7366 |
|  | 76->84 | 0.41309 |  |  |  |  |  |
|  | 80->85 | 0.29687 |  |  |  |  |  |
|  | 81->85 | 0.23817 |  |  |  |  |  |
| 16 | 79->86 | 0.26027 | 6.4155 | 193.26 | 0.0075 | -8.7526 | -8.4985 |
|  | 79->87 | 0.22835 |  |  |  |  |  |
|  | 80->85 | 0.42588 |  |  |  |  |  |
| 17 | 76->84 | 0.38503 | 6.4533 | 192.12 | 0.0071 | -11.6153 | -12.5441 |
|  | 80->85 | -0.25396 |  |  |  |  |  |
| 18 | 78->85 | 0.22849 | 6.4841 | 191.21 | 0.0609 | -16.8069 | -18.5293 |
|  | 83->88 | 0.2501 |  |  |  |  |  |
|  | 83->89 | 0.42866 |  |  |  |  |  |
|  | 83->90 | -0.28733 |  |  |  |  |  |
| 19 | 75->84 | 0.38178 | 6.5450 | 189.43 | 0.0309 | 0.4248 | -1.7299 |
|  | 79->86 | 0.29467 |  |  |  |  |  |
|  | 79->87 | 0.22804 |  |  |  |  |  |
| 20 | 81->86 | 0.23843 | 6.7084 | 184.82 | 0.1948 | 8.7097 | 8.3076 |
|  | 82->86 | 0.52215 |  |  |  |  |  |
| 21 | 78->85 | 0.51738 | 6.7553 | 183.54 | 0.1769 | 65.1155 | 66.5177 |
| 22 | 73->84 | 0.48991 | 6.8979 | 179.74 | 0.0892 | -76.508 | -78.8231 |
|  | 75->84 | -0.29786 |  |  |  |  |  |
| 23 | 76->85 | 0.26282 | 7.0644 | 175.51 | 0.0384 | 28.1311 | 30.9302 |
|  | 77->85 | 0.28877 |  |  |  |  |  |
|  | 82->87 | -0.22757 |  |  |  |  |  |
| 24 | 83->89 | 0.4145 | 7.1308 | 173.87 | 0.0073 | -6.0227 | -6.4917 |
|  | 83->90 | 0.42482 |  |  |  |  |  |
|  | 83->92 | -0.2403 |  |  |  |  |  |
| 25 | 71->84 | -0.29868 | 7.2280 | 171.53 | 0.0106 | 3.1044 | 2.3001 |
|  | 79->85 | 0.51217 |  |  |  |  |  |
| 26 | 77->85 | -0.2558 | 7.2508 | 170.99 | 0.1068 | 31.8482 | 33.4586 |
|  | 80->86 | -0.23481 |  |  |  |  |  |
|  | 81->86 | 0.3201 |  |  |  |  |  |
| 27 | 71->84 | -0.35102 | 7.3060 | 169.70 | 0.0975 | 1.809 | 2.0274 |
|  | 76->85 | 0.22398 |  |  |  |  |  |
|  | 79->85 | -0.22748 |  |  |  |  |  |
|  | 82->87 | 0.36354 |  |  |  |  |  |
| 28 | 77->85 | 0.39747 | 7.3238 | 169.29 | 0.0518 | -14.3708 | -15.6111 |
|  | 81->86 | 0.24189 |  |  |  |  |  |
| 29 | 71->84 | 0.33118 | 7.3807 | 167.99 | 0.0122 | 8.1154 | 8.8995 |
|  | 74->84 | -0.24789 |  |  |  |  |  |
|  | 79->85 | 0.29006 |  |  |  |  |  |
|  | 82->87 | 0.26738 |  |  |  |  |  |
| 30 | 74->84 | 0.37732 | 7.4827 | 165.69 | 0.0287 | -1.3581 | -1.8469 |
|  | 76->85 | 0.25938 |  |  |  |  |  |
| 31 | 83->90 | 0.22836 | 7.5433 | 164.36 | 0.0055 | -2.3226 | -3.8285 |
|  | 83->92 | 0.49899 |  |  |  |  |  |
| 32 | 82->88 | -0.23796 | 7.5584 | 164.03 | 0.0167 | -6.381 | -7.7184 |
|  | 83->92 | 0.28632 |  |  |  |  |  |
| 33 | 83->91 | 0.54848 | 7.5913 | 163.32 | 0.0084 | -0.0524 | 0.4203 |
|  | 83->93 | -0.32471 |  |  |  |  |  |
| 34 | 75->85 | 0.38485 | 7.6044 | 163.04 | 0.0224 | 8.8849 | 7.6913 |
|  | 76->85 | -0.24328 |  |  |  |  |  |
|  | 81->87 | -0.31327 |  |  |  |  |  |
|  | 82->87 | 0.2276 |  |  |  |  |  |
| 35 | 75->85 | 0.38703 | 7.6510 | 162.05 | 0.0158 | 9.1215 | 9.8447 |
|  | 81->87 | 0.38004 |  |  |  |  |  |
| 36 | 72->84 | 0.60516 | 7.7474 | 160.03 | 0.0420 | 34.2985 | 36.5116 |

*^a^*Number of the excited states; *^b^*Only transitions with contribution over 10.0% were listed; *^c^*Configuration-interaction coefficient; *^d^*Excitation energy; *^e^*Wavelength; *^f^*Oscillator strength; *^g^*Rotatory strength in velocity form (10^-40^ cgs); *^h^*Rotatory strength in length form (10^-40^ cgs).
